# The impact of the administration of red ginseng (*Panax ginseng*) on lipid metabolism and free fatty acid profiles in healthy horses using a molecular networking approach

**DOI:** 10.3389/fvets.2024.1285000

**Published:** 2024-01-24

**Authors:** Young Beom Kwak, Hye Hyun Yoo, Jungho Yoon

**Affiliations:** ^1^Racing Laboratory, Korea Racing Authority, Jeju, Republic of Korea; ^2^Institute of Pharmaceutical Science and Technology and College of Pharmacy, Hanyang University, Ansan, Republic of Korea; ^3^Equine Referral Clinic, Jeju Stud Farm, Korea Racing Authority, Jeju, Republic of Korea

**Keywords:** free fatty acids, horses, lipid metabolism, molecular networking, red ginseng

## Abstract

This study investigated the potential benefits of the administration of red ginseng (RG) on lipid metabolism and the profiles of individual free fatty acids (FFAs) in healthy horses. Eight healthy horses, raised under similar conditions, were randomly divided into two groups, each comprising four horses. The experimental group received powdered RG (600 mg/kg/day) mixed with a carrier, and the control group received only the carrier. The parameters associated with lipid metabolism and probable adverse effects were evaluated in both groups after 3 weeks. The computational molecular networking (MN) approach was applied to analyze the FFA profiles. The results indicated that RG administration significantly reduced blood triglyceride levels in the experimental group. Analysis of the FFAs using MN revealed significant decreases in specific types of FFAs (C12:0, dodecanoic acid; C14:0, myristric acid; C18:1, oleic acid; C18:2, linoleic acid). RG consumption did not produce significant adverse effects on the renal, hepatic, and immune functions. Thus, RG was found to effectively modulate lipid metabolism and the levels of individual FFAs. The application of the MN for the analysis of FFAs represents a novel approach and can be considered for future research.

## Introduction

Red ginseng (RG; heat-processed product of *Panax ginseng*, C.A. Meyer) is a herbal medicine widely used in Eastern Asia for thousands of years (1, 2). The popularity of RG as a herbal supplement in complementary and alternative medicine has grown in Western countries over the last few decades (3, 4). Ginsenosides and gintonin, the major pharmacologically active components of RG, produce diverse effects in the various tissues, which make the overall activity of RG very complex (1, 3). The pharmacological and biological properties of RG include anti-inflammatory, antioxidant, antitumor, antidiabetic, and antistress activities. These properties have shown benefits in cardiovascular, liver, and neurological diseases, and in improving metabolic parameters, including lipid metabolism (2, 3, 5–8).

RG is a common herbal extract used as a feed additive and alternative medicine in equines (9). Over the past five years, in doping inspections for feed additives used in racehorses in the Jeju region of Korea, RG has accounted for a significant share of 34.7% (76 out of 219 total feed additive inspections). Despite the extensive utilization of RG in the equine marketplace for purposes such as growth enhancement, disease prevention, stress amelioration, and performance improvement, there is little scientific evidence regarding its use in horses (9, 10). Thus, most of the evidence on the effects of RG in horses is anecdotal. Therefore, investigation on the use, benefits, and safety of RG in equines may help dispel concerns over its usage and enable horse owners and veterinarians to make a more justifiable choice.

The administration of ginseng to horses is generally prohibited in both the International Federation of Horseracing Authorities (IFHA) and the International Equestrian Federation (FEI) regulations for events such as racing and equestrian competitions (11). However, rules and regulations pertaining to the use of herbal products can vary significantly depending on the specific racing and equestrian authorities. For instance, British rules of horseracing state that all feed supplements or medications, including herbal extracts, can be freely given to horses during training but must be discontinued several days prior to a race for preventive measures (9).

Triglycerides (TG) are esters derived from glycerol and three fatty acids, constituting the primary components of body fat in animals (12). Free fatty acids (FFAs) are released from adipose tissue via the lipolysis of TG and circulate in the plasma (13). Lipolysis, the breakdown of TG into FFAs, is a physiologically important process that meets the energy demands of the body (14). Epidemiological analysis and clinical trials in humans have shown that elevated levels of TG and FFAs are associated with various metabolic and cardiovascular diseases that increase all-cause mortality (15). These disease states include insulin resistance, fatty liver, pancreatitis, arterial hypertension, atherosclerosis, and myocardial dysfunction (15–21).

FFAs are important biological molecules that serve as a major energy source and are key components of biological membranes, with each type of FFA exerting a distinct effect on physiological processes (22, 23). In addition, FFAs play important roles in metabolic regulation and altered plasma FFA concentrations have the potential to contribute to the development and progression of various metabolic disorders (22, 23). Therefore, the profiling of alterations in specific FFAs in the plasma of individuals holds significant importance. Until recently, due to the diversity in FFAs and the methodological limitations of FFA screening, which demands considerable time and resources, the profiling of FFAs has been technically challenging. However, with the rapid development of omics and artificial intelligence in molecular networking (MN) using the Global Natural Product Social Molecular Networking platform (GNPS^1^), an alternative approach for screening unknown substances based on the pattern similarity of spectra in mass spectrometry (MS), is now possible (24, 25). This approach offers the advantages of speed and cost-effectiveness, and it is believed that MN analysis can be applied to FFA profiling in horses.

In this study, we evaluated the safety of RG administration in horses and quantitatively investigated its impact on lipid metabolism. Additionally, we applied a computational MN strategy to analyze the changes in the fatty acid profile. To the best of our knowledge, this is the very first investigation providing evidence of RG’s effectiveness in modulating TG and FFAs in horses through the application of the MN strategy.

## Materials and methods

### RG information

The RG powder was manufactured by the Pocheon Ginseng Farming Association using 6-year-old Korean ginseng (Korea Red Ginseng Powder Gold, product reporting number 200600151445 by the Ministry of Food and Drug Safety in the Republic of Korea). As per the manufacturer’s instructions, RG was prepared by steaming ginseng at 95°C for 3 h and subsequently drying it at 30°C for 30 h. The dried ginseng was then ground to produce the powder. The RG powder contained a total ginsenoside content of 19.65 mg/g. The analysis methods and results of ginsenoside components within the RG product used in this study are presented in the Supplementary Table S1.

### Quantitative measurement of RG components in plasma

The methods for the analysis of ginsenosides and the validation procedures for quantitative analysis are described in a previously published paper (26).

### Horses and experimental design

For the experiment, eight adult thoroughbred riding horses, aged 3–20 years and weighing 480 ± 20 kg, were randomly divided into control and experimental groups. All the horses involved in the experiment were raised and managed under the same conditions for at least 1 year on a farm. The horses in the experimental group were fed ground RG (600 mg/kg/day) in a carrier of molasses and water for 3 weeks. The horses in the control group received the carrier alone. All horses received water and feed *ad libitum* under the same conditions during the experiment. The animal protocols for this study were approved by the Institutional Animal Care and Use Committee of the Korea Racing Authority (KRA IACUC-2203-AEC-2203).

### Hematological and blood biochemistry analyses

Hematological and biochemical analyses were performed to evaluate the efficacy and safety of RG. Blood samples were collected in three types of blood collection tubes: serum separator tube for blood biochemistry analysis, ethylenediaminetetraacetic acid tube for white blood cell count, and heparin tube for FFA and MN analysis (BD Vacutainer® blood collection tubes, Becton-Dickinson and Company, Franklin Lakes, NJ, USA). Venipuncture was performed in the morning after the RG feeding. Laboratory analysis of blood biochemistry for parameters associated with lipid metabolism, and biomarkers of renal, hepatic, and immunological functions were performed (GreenVet laboratory, Yongin, Korea).

### Molecular networking analysis

A molecular networking method was used to screen FFAs in horse plasma. Raw data in AIA format (.CDF files) were obtained from Agilent Technologies Enhanced Data Analysis (Version D 00.00.38) and processed using MZmine 2 (version 2.53) software. The process involved mass detection, ion-specific chromatogram creation, chromatogram deconvolution, and spectral deconvolution using the Hierarchical Clustering method. Peak lists were adjusted for samples using the ADAP alignment module and exported to data files (.mgf and _quant.csv). The molecular networking was performed in GNPS with specific settings, including fragment ion mass tolerance, minimum matched peaks, and cosine value thresholds. Molecular identification was done using the NIST14 MS database (NIST MS Search 2.3). Finally, Cytoscape 3.7.2 software was used to visualize and annotate the molecular networking data. The screening data served as the fundamental data for the preparation of standard materials for FFAs quantification and were validated by comparing them with the spectra of prepared standard materials.

### Quantitative analysis of FFAs using GC–MS

The analytical method and quantitative validation procedures of FFAs analysis using GC–MS have been provided in the Supplementary materials and modified by previous studies (27–31).

### Statistical analysis

The parameters in this study were validated using the paired samples t-test with the Jamovi software. *p* values less than 0.05 were considered statistically significant. All data were expressed as the mean ± SD (*n* = 4).

## Results

### Quantitative measurement of RG components in plasma

In the group of horses (*n* = 4) that were fed RG, four ginsenosides were detected in the plasma (Rb1; 125.1 ± 79.3, Rb2; 57.7 ± 25.0, Rc; 73.9 ± 40.0, Rd.; 32.8 ± 10.2, mean ± SD), which were absent in the control group. Ginsenosides are glycosides that contain an aglycone with a dammarane skeleton and exhibit various biological activities (32). Over the course of 3 weeks in the experiment, horses were administered RG, and it was imperative to verify the presence of RG constituents in the body and bloodstream of the experimental group, distinguishing them from the control group. Ginsenosides, recognized as the major active compounds in RG with documented effects on the body in many studies (33, 34), were employed as indicators. The detection of ginsenosides allowed us to infer the distribution of RG components in the experimental group’s body.

### RG lowers blood triglyceride levels

**Figure 1:**
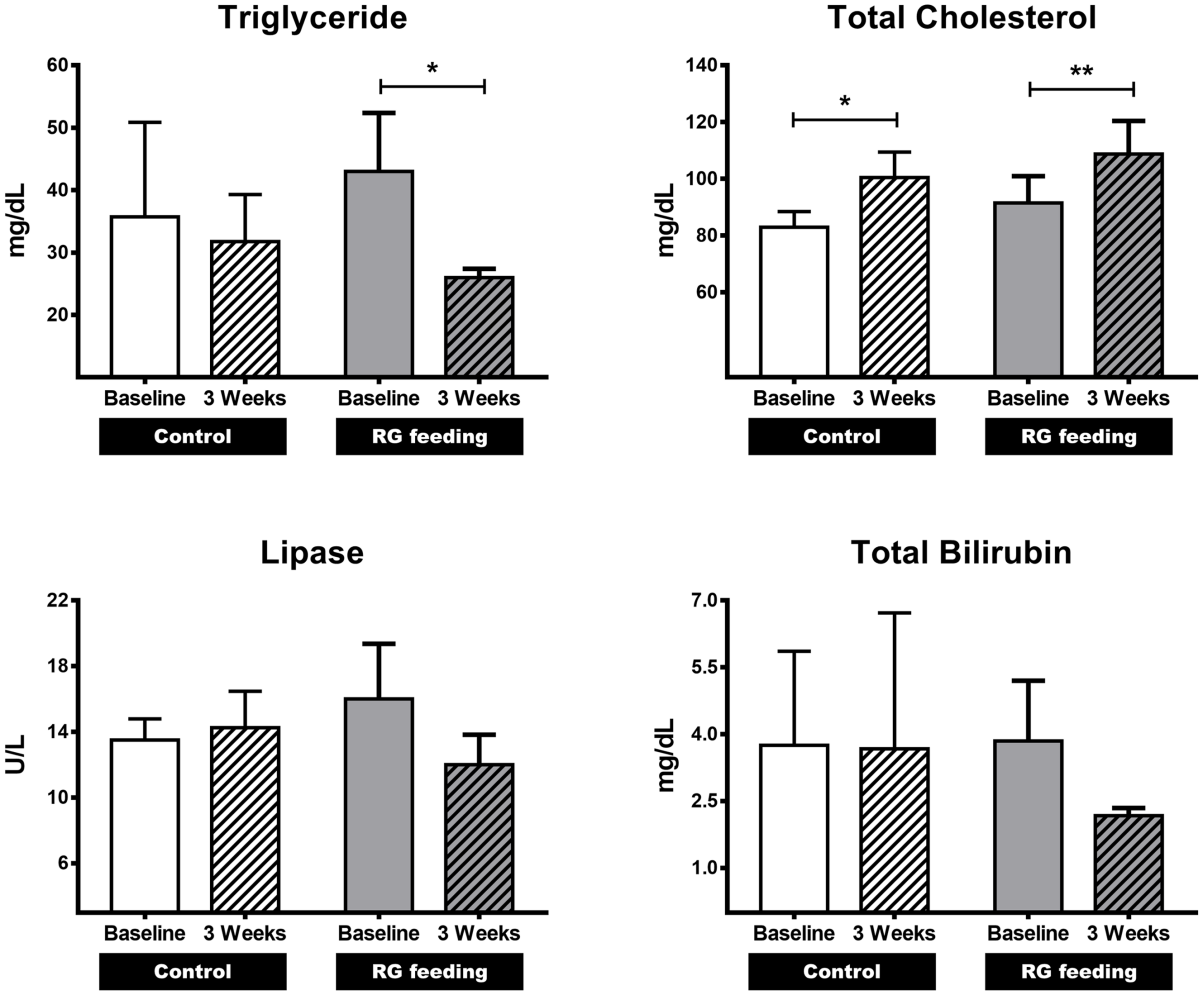
Effect of red ginseng (RG) on lipid metabolism in horses. The impact of RG consumption on lipid metabolism was investigated by analyzing four variables: triglycerides (TG), total cholesterol (TC), lipase, and total bilirubin (TB). Among these variables, the TG level showed a significant decrease following RG feeding. *p < 0.05; **p < 0.01.

To determine the effects of RG on the parameters associated with lipid metabolism, the levels of TG, total cholesterol (TC), lipase, and total bilirubin (TB) were assessed after 3 weeks of RG administration. In the RG-fed group, the TG levels significantly decreased (*p* < 0.05) from 43.0 ± 9.38 to 26.0 ± 1.41 mg/dL compared to the control group (from 35.8 ± 15.13 to 31.8 ± 7.54) after 3 weeks (Figure 1). The lipase and TB levels also decreased, from 16.0 ± 3.37 to 12.0 ± 1.83 U/L and from 3.85 ± 1.348 to 2.17 ± 0.171 mg/dL, respectively, but these differences were not statistically significant (Figure 1). There was a statistically significant increase in TC levels in both the RG-fed and control groups (from 83.0 ± 5.48 to 100.5 ± 8.96 mg/dL in control; from 91.5 ± 9.47 to 108.8 ± 11.67 mg/dL in RG-fed group) (Figure 1).

### Molecular networking screening

**Figure 2:**
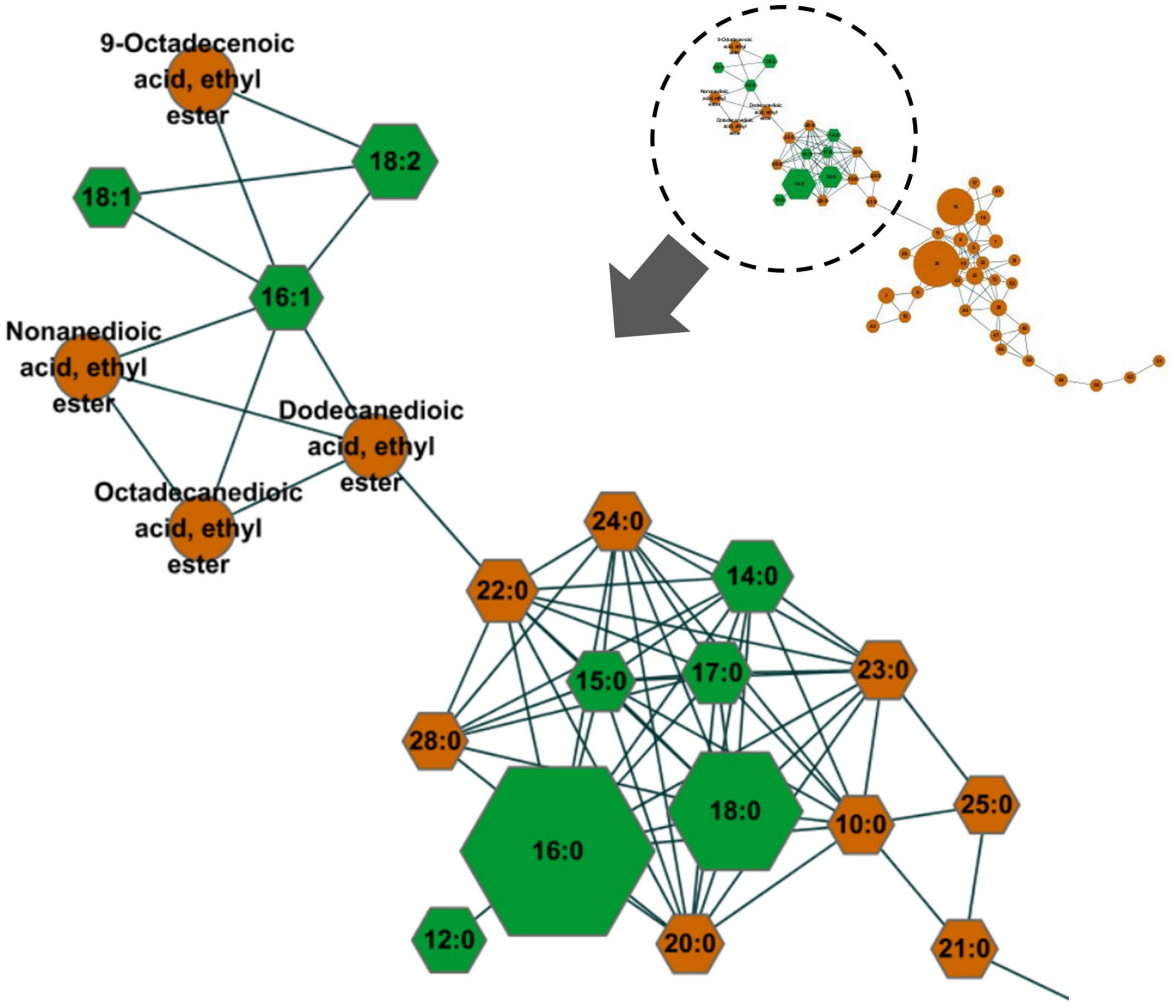
Results of the molecular networking analysis of gas chromatography–mass spectrometry (GC–MS) data were used to screen for free fatty acids (FFAs) in equine plasma. The 17 nodes of detected FFAs were indicated using hexagonal shapes. The size of the nodes was proportionally applied to the area of the analysis signals. The nine FFAs (C12:0, C14:0, C15:0, C16:0, C17:0, C18:0, C16:1, C18:1, C18:2; green hexagonal shapes), which had levels above the lower limit of quantification (LLOQ), were quantified to profile the FFAs. The levels of the other FFAs were below the LLOQ. The spectral similarities among these FFAs formed a connectivity group, enabling quick and selective screening within the molecular network.

The MN strategy was used to screen for the type of FFAs in horse plasma. MN revealed a total of 55 nodes, with FFAs comprising 14 saturated fatty acids (capric acid, C10:0; dodecanoic acid, C12:0; myristic acid, C14:0; pentadecanoic acid, C15:0; palmitic acid, C16:0; hepadecanoic acid, C17:0; stearic acid, C18:0; arachidic acid, C20:0; heneicosanoic acid, C21:0; behenic acid, C22:0; tricosanoic acid, C23:0; lignoceric acid, C24:0; pentaosanoic acid, C25:0; montanic acid, C28:0) and 3 unsaturated fatty acids (palmitoleic acid, C16:1; oleic acid, C18:1; linoleic acid, C18:2) (Figure 2). Other nodes included 9-octadecenoic acid ethyl ester, nonanedioic acid ethyl ester, dodecanedioic acid ethyl ester, octadecanedioic acid, ethyl ester, biocytin, *N*-acetylmannosamine, aminoadipic acid, among others.

### Quantitative analysis of FFAs

**Figure 3:**
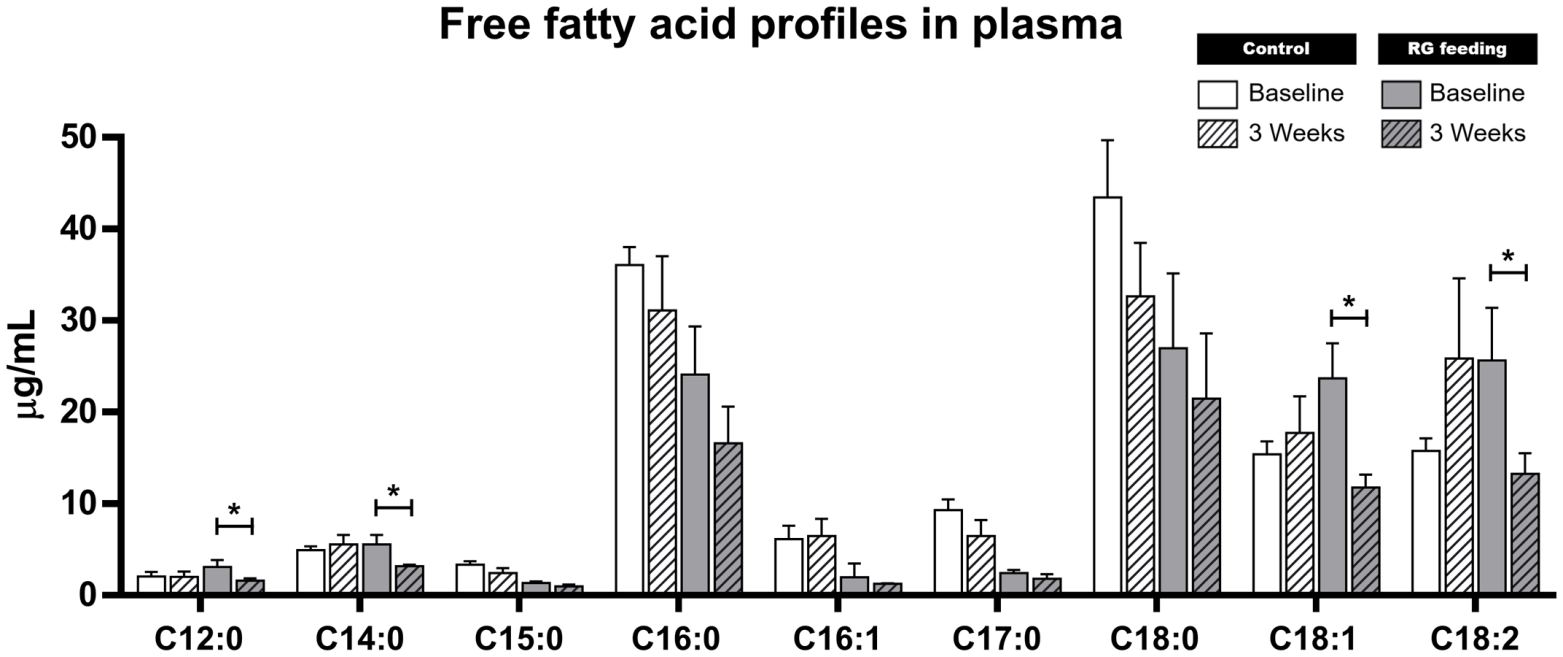
Changes in free fatty acid (FFA) profiles following red ginseng (RG) feeding. Out of the nine FFAs screened using computational molecular networking (MN), there were significant decreases in the levels of four FFAs (C12:0, C14:0, C18:1, C18:2) after 3 weeks of RG consumption. *p < 0.05. C12:0, dodecanoic acid; C14:0, myristric acid; C15:0, pentadecanoic acid; C16:0, palmitic acid; C16:1, palmitoleic acid; C17:0, hepadecanoic acid; C18:0, stearic acid; C18:1, oleic acid; C18:2, linoleic acid.

Quantitative analysis using GC–MS was conducted after preparing standard substances of confirmed free fatty acids. As a result, six saturated fatty acids (C12:0, C14:0, C15:0, C16:0, C17:0, C18:0) and three unsaturated fatty acids (C16:1, C18:1, C18:2) were quantitatively measurable in equine plasma due to signals exceeding the lower limit of quantification. As a result, after consumption of RG, there was a significant decrease in the levels of four FFAs (C12:0, C14:0, C18:1, C18:2) (*p* < 0.05, from 3.11 ± 1.27 to 1.60 ± 0.41 μg/mL, from 5.57 ± 1.78 to 3.19 ± 0.31 μg/mL, from 23.68 ± 6.67 to 11.77 ± 2.47 μg/mL, and from 25.64 ± 10.00 to 13.28 ± 3.86 μg/mL, respectively) in the RG-fed group (Figure 3). While there was an overall decrease observed in the levels of the other types of FFAs after RG feeding, statistical significance was not evident (Figure 3). These results contrasted with those of the control group.

### Short-term safety evaluation of RG in horses

To assess the safety of RG in horses, the biological markers for renal function (blood urea nitrogen [BUN], creatinine [CREA]), hepatic function (aspartate aminotransferase [AST], gamma-glutamyl transferase [GGT], alkaline phosphatase [ALP], and albumin, [ALB]), and immunological changes (white blood cells [WBC], and serum amyloid A [SAA]) were determined (Table 1). Among the eight parameters, the mean levels of four parameters (ALP, BUN, CREA, and WBC) decreased and the mean levels of two parameters (AST and GGT) increased in the RG-fed group compared to those of the control group. However, there were no statistically significant differences in the parameters between the control and RG-fed groups (Table 1).

**Table 1:** Short-term safety assessment of red ginseng (RG) (*n* = 4, mean ± SD).

Groups	Liver				Kidney		Inflammation	
	AST (g/dL)	GGT (U/L)	ALP (U/L)	ALB (g/dL)	BUN (mg/dL)	CREA (mg/dL)	WBC (K/μL)	SAA (mg/L)
**Control**	226.75 ± 17.90	11.00 ± 1.41	127.75 ± 15.24	3.38 ± 0.25	16.50 ± 2.65	1.90 ± 0.50	6.88 ± 1.12	< 10
**RG feeding**	228.50 ± 55.12	13.00 ± 3.92	120.00 ± 24.82	3.38 ± 0.19	14.00 ± 2.16	1.57 ± 0.24	4.96 ± 3.44	< 10
***P* value**	0.962	0.415	0.552	1	0.278	0.205	0.315	-

No statistically significant differences were observed between the control and RG-fed groups for the analyzed parameters. AST, aspartate aminotransferase; GGT, gamma-glutamyl transferase; ALP, alkaline phosphatase; ALB, albumin; BUN, blood urea nitrogen; CREA, creatinine; WBC, white blood cell; SAA, serum amyloid A.

## Discussion

This study describes the potential benefits of RG administration to healthy horses with respect to lipid metabolism and discusses its short-term safety. Several earlier studies have focused on the role of RG supplementation on lipid metabolism in various animals (3–5, 35). However, few studies on the effects of RG on the lipid profile and safety in horses have been conducted so far. Considering that individual responses to RG may vary depending on the animal species and specific health conditions, further research on RG’s effects and safety in horses was warranted to ensure the responsible and effective use of RG as an herbal supplement in complementary and alternative medicine.

In this study, we analyzed the effects of RG administration on parameters associated with lipid metabolism and short-term safety in horses. After 3 weeks of RG administration (600 mg/kg) the major components of RG (Rb1, Rb2, Rc, and Rd) were confirmed in the plasma of the RG-fed horses. This step of the experiment indicates that the biologically active components of RG were available for action. It is known that approximately 150 ginsenosides have been identified to date and 35 ginsenosides are major components in white ginseng or RG (32). Depending on their aglycone moieties, ginsenosides are divided into (20s)-protopanaxadiol (PPD, including Rb1, Rb2, Rc, Rd., Rg3, F2, and Rh2) and (20s)-protopanaxatriol (PPT, including Rg1, Rh1, and Re) (33, 36). It is known that both Rb1 and PPT-type ginsenosides influence the expression of peroxisome proliferator-activated receptor (PPAR) in the liver, inhibiting fat accumulation (37–39). PPD-type ginsenosides have been reported to inhibit the activity of pancreatic lipase and promote fat excretion (40). Previous studies conducted on humans and rodents have shown that RG reduces the circulating levels of TG and FFAs and improves metabolic disorders (4, 5, 32, 41). The results of this study in horses also showed that RG administration decreased the levels of TG and FFAs. However, no statistically significant changes were observed in the TC, lipase, and TB levels. This could potentially be attributed to the fact that the experiment in this study targeted healthy horses under normal dietary conditions, unlike previous research that often focused on obese models. Further experiments with diseased animal models with dietary changes are needed to investigate the effect of RG on these three parameters.

RG and its components regulate lipid metabolism through various processes. The liver, a key organ in synthesizing TG for energy storage, plays a crucial role in these changes (42–46). In the liver, RG and its components activate adenosine monophosphate-activated kinase (AMPK) and PPAR pathways, leading to the inhibition of TG synthesis and fat accumulation through related enzymes such as FA synthase, 3-hydroxy-3-methyl-glutaryl-coenzyme A reductase, and phosphoenolpyruvate carboxykinase (34, 37–39, 42–47). In digestion and absorption, ginsenosides can regulate differentiation of adipocytes. They act as pancreatic lipase inhibitors, promoting the excretion of fat through feces, thus preventing the intra-body accumulation of lipids and increasing fecal weight and lipid content (48).

However, the intricate mechanisms between RG and lipid metabolism require a deeper understanding. Specifically, the physiological mechanisms of RG in equines remain unexplored territory, signifying the necessity for further extensive research in the future. With the advancement of systems biology, multi-omics analyses suggested that ginseng metabolites down-regulate genes associated with lipid metabolism and modulate the synthesis and decomposition of phosphatidylcholine in the glycerophospholipid (GPL) species through gut microbiota in rodents (49, 50). Furthermore, the sphingolipid and GPL, which show bidirectional homeostatic crosstalk between them and contribute to metabolic diseases, were influenced after RG feeding, impacting the regulation of lipid metabolism (51–54). In this regard, exploring research directions, including omics, to deepen understanding of the mechanisms associated with RG and lipid metabolism appears crucial for the future.

Research on plants or their extracts as feed additives has predominantly been conducted in species other than equines (9). Although the use of plant extracts is theoretically safer than synthetic drugs, it is essential to conduct comprehensive evaluations for potential negative side effects, optimal dosage, duration of administration, as well as potential herb-drug interactions to address concerns over the use of herbal extracts in equines. Hence, this study attempted to evaluate the safety of oral RG feeding at a dose of 600 mg/kg in horses by analyzing the markers for renal/hepatic function and inflammation. The results showed no significant difference between the control and RG-fed groups, suggesting that short-term oral resource-intensive administration of RG does not result in notable adverse effects on the kidneys, liver, and immune system. Investigations into the optimal dosages, durations, route of administration, potential cumulative effects, and herb-drug interactions are warranted for the appropriate and responsible use of RG in the equine industry.

RG has been used as complementary and alternative medicine for thousands of years in Eastern countries (1). However, there are still no guidelines regarding the optimal dosage and administration duration. Previous human studies that evaluated the clinical effect of RG varied the dosage from 100 to 600 mg/kg, and the administration duration ranged from 1 day to 27 months (55). In studies describing herb-drug interactions, a commonly observed administration duration ranged from 2 to 4 weeks (56). Considering the previous studies and available resources for our experiment, we orally administered RG to horses at a dose of 600 mg/kg for a duration of 3 weeks. However, further research is essential to analyze various responses and effects of RG based on its concentration and administration duration.

Individual FFAs vary in terms of chain length and saturation and are altered in response to metabolic changes (57). Variations in the circulating levels of specific FFAs exert distinct pathobiological effects and are important for disease prognosis (22, 23). The computational MN analysis conducted using the GNPS has opened up new horizons for the analysis of unknown substances, enabling a rapid and cost-effective approach that was previously impossible. Using the MN analysis approach, we additionally semi-quantified the levels of different individual FFAs after RG consumption and analyzed their structural similarity, thus identifying the potential significance of the circulating FFA species (Figures 2, 3). It is worth noting that this study was the first to use the MN approach for FFA screening and analysis in horses. The MN approach can be applied not only in this study but also in various other research fields, not limited only to equines.

FFAs play a crucial role in the pathogenesis of metabolic disorders and therapeutic approaches for modulating FFA metabolism hold promise for the patients with these diseases (58, 59). While there are drugs available that reduce circulating FFAs or interfere with their oxidation, concerns about the efficacy and safety of these drugs have led to the need for new drug targets or alternative therapies (58, 59). As each type of FFA shows distinct effects on the various physiological and disease processes (22, 23), understanding the individual changes in the FFAs and utilizing them for disease treatment is warranted. Furthermore, in experiments conducted on humans and mice, there is evidence of the anti-fatigue effects and changes through lipid metabolism following ginseng administration in response to exercise (60, 61). However, there is a lack of research specifically addressing the association with individual FFAs. As described in this study, RG supplementation resulted in the modification of the blood FFA profile in horses (Figure 3). This indicates that it is necessary to elucidate the specific effects of each FFA on the treatment of metabolic diseases and the anti-fatigue effects after exercise following RG administration in future research.

This study is the first research to analyze the changes induced by RG in individual FFAs using MN approach. However, this study has some limitations. Conducting experiments using horses is costly and resource-intensive compared to other animal species. Due to the limited number of horses used in this study, there are some differences in the baseline data of control and RG-fed horses regarding the parameters of lipid metabolism and FFAs (Figures 1, 3). However, based on statistical analysis, these differences on lipid metabolism parameters were not significant (*p* > 0.05) (Figure 1). The significant reduction observed in four FFAs (C12:0, C14:0, C18:1, C18:2) after RG administration highlights the modulation of these four FFAs by RG, considering that these FFAs tended to increase when RG was not administered (Figure 3). For the other FFAs (C15:0, C16:0, C16:1, C17:0, C18:0) that did not show significant changes after RG administration, there were no notable changes even when RG was not administered, and similar patterns of change were observed.

## Conclusion

In conclusion, we evaluated the short-term safety and effects of RG administration on lipid metabolism in healthy horses. Additionally, we quantified the levels of individual FFAs and analyzed the structural similarities using MN to identify the changes in the specific types of FFAs. This is the first study to assess the effects of RG on lipid metabolism in horses and provides evidence of the effectiveness of RG in reducing TG and FFAs. Furthermore, we present a novel approach for the rapid and efficient analysis of FFAs through a computational MN methodology. These findings contribute to the existing knowledge base on the use of RG as an alternative medicine and nutritional supplement for horses.

## Data availability statement

The original contributions presented in the study are included in the article/Supplementary material, further inquiries can be directed to the corresponding author.

## Ethics statement

The animal study was approved by Institutional Animal Care and Use Committee of the Korea Racing Authority. The study was conducted in accordance with the local legislation and institutional requirements.

## Author contributions

YK: Conceptualization, Data curation, Formal analysis, Methodology, Resources, Software, Visualization, Writing – original draft, Writing – review & editing. HY: Conceptualization, Data curation, Formal analysis, Methodology, Resources, Validation, Writing – review & editing. JY: Conceptualization, Data curation, Formal analysis, Methodology, Resources, Software, Supervision, Visualization, Writing – original draft, Writing – review & editing.

## Footnotes

^1^ https://gnps.ucsd.edu/ProteoSAFe/static/gnps-splash.jsp
