# Supplementary material for: The impact of the administration of red ginseng (Panax ginseng) on lipid metabolism and free fatty acid profiles in healthy horses using a molecular networking approach
Source: Front Vet Sci. 2024 Jan 24;11:1285000. doi: 10.3389/fvets.2024.1285000 (PMC10851614; doi:10.3389/fvets.2024.1285000)
Supplement: Supplementary file 1 [file Data_Sheet_1.docx]

**Supplementary data**

**Materials**

The material standard of ginsenosides (>98%) for analysis were obtained from Ambo institute (Daejeon, Korea). Dodecanoic acid (12:0), myristric acid (14:0), pentadecanoic acid (15:0), palmitic acid (16:0), palmitoleic acid (16:1), hepadecanoic acid (17:0), stearic acid (18:0), oleic acid (18:1), linoleic acid (18:2), arachidic acid (20:0), heneicosanoic acid (21:0), behenic acid (22:0), tricosanoic acid (23:0), lignoceric acid (24:0), palmitic acid-d31 (16:0, d31), digitoxin, and formic acid were obtained from Sigma-Aldrich (St. Louis, MO, USA), and materials were stored at -20°C until used. The purity of standard materials was used as analytical standard. MethPrep II was obtained from Alltech Inc. (Deerfield, IL). Hexane was purchased from Junsei Chem (Chou-ku, Japan). Chloroform, methanol, and distilled water were purchased from J.T. Baker (Phillipsburg, NJ, USA). All solvent was used as HPLC-grade quality

**The analysis of ginsenoside content within the red ginseng product**

The method for measuring red ginseng powder was conducted by referring to the procedures outlined in a previously published paper (1).

**Sample preparation and GC-MS analysis**

Fatty acid and internal standard (ISTD) stock solutions were prepared in hexane:chloroform (1:1 or 1:3, v/v) at 1 mg/L, stored at -20°C. The ISTD (palmitic acid-d31) was diluted to a working standard at 500 µg/mL in hexane:chloroform (1:1, v/v). Other stock solutions were diluted similarly and mixed with fatty acids to create working standards from lower limit of quantification (LLOQ) to 100 µg/mL. Plasma, left at room temperature for 30 min, was extracted following the Folch method (2). After adding 40 µL ISTD and 10 ml Folch reagent to 1000 µL plasma, samples were shaken for 10 min, centrifuged, and the chloroform layer was retained. Dried residues were reconstituted for GC-MS analysis (HP-ULTRA 2 column, Agilent 6890N). Gas chromatography (260˚C injector, splitless mode) included sample washing with methanol, using helium as the carrier gas. The temperature gradient started at 80°C, increased to 150°C at 30°C/min, then to 550°C at 10°C/min, followed by an increase to 310°C at 30°C/min and a 3.5 min hold, totaling 18.83 minutes. Detection used an Agilent Technologies 5973N in full scan mode (range *m/z* 50–450) for molecular networking and SIM mode for quantitative analysis at 300°C. The mean recovery of FFAs was 99.8% (SD = 5.1%).

**Method validation for equine plasma analysis**

We conducted an analytical method validation by assessing the LLOQ, linearity, precision, and accuracy. Linearity was evaluated through the construction of calibration curves, utilizing the peak area ratios of free fatty acids at five spiked concentrations to the peak area of the internal standard (palmitic acid-d31). LLOQ was determined as the minimum concentration with a Signal-to-Noise ratio exceeding 9. Precision and accuracy were evaluated by analyzing quality control samples at a concentration of 30 μg/mL (n=2).

**Reference**

1. Kim JK, Choi MS, Jeung W, Ra J, Yoo HH, Kim DH. Effects of Gut Microbiota on the Pharmacokinetics of Protopanaxadiol Ginsenosides Rd, Rg3, F2, and Compound K in Healthy Volunteers Treated Orally with Red Ginseng. *J Ginseng Res* (2020) 44(4):611-8. doi: 10.1016/j.jgr.2019.05.012.

2. Folch J, Lees M, Sloane Stanley GH. A Simple Method for the Isolation and Purification of Total Lipides from Animal Tissues. *J Biol Chem* (1957) 226(1):497-509.

Table S1. The analysis results of ginsenoside content within the RG product (Korea Red Ginseng Powder Gold)

| Component | Concentration (mg/g) | Component | Concentration (mg/g) |
| --- | --- | --- | --- |
| Rg1 | 4.38 | F1 | 0.68 |
| Re | 5.53 | Rd | 0.97 |
| Rf | 1.60 | F2 | 0.40 |
| Rb1 | 0.34 | Rg3 | 0.20 |
| Rg2 | 0.59 | CK | 0.87 |
| Rc | 1.05 | Rh2 | 0.21 |
| Rb2 | 2.83 |  |  |

| Analyte | Monitoring ion  in SIM mode (*m/z*) | LLOQ (μg/mL) | *Range (μg/mL) | Linearity (R^2^) | Precision  (%) | Accuracy  (RSD) |
| --- | --- | --- | --- | --- | --- | --- |
| C12:0 | 74 | 0.5 | 0.5–100 | 0.9842 | 4.8 | 101.9 |
| C14:0 | 74 | 1 | 1–100 | 0.9802 | 0.9 | 106.0 |
| C15:0 | 74 | 0.5 | 0.5–100 | 0.9772 | 1.6 | 93.1 |
| C16:0 | 74 | 5 | 5–100 | 0.9676 | 0.2 | 101.2 |
| C17:0 | 74 | 1 | 1–100 | 0.9443 | 1.9 | 98.1 |
| C18:0 | 74 | 1 | 1–100 | 0.9448 | 2.7 | 104.2 |
| C16:1 | 55 | 1 | 1–100 | 0.9825 | 3.6 | 98.6 |
| C18:1 | 55 | 5 | 5–100 | 0.9730 | 3.3 | 97.5 |
| C18:2 | 67 | 5 | 5–100 | 0.9795 | 3.3 | 99.7 |

Table S2. The validation result of quantitative analysis for Free Fatty Acids (FFAs)

* When LLOQ is 0.5 μg/mL, the quantification range is set at 0.5, 10, 20, 50, and 100 μg/mL. when it is 1 μg/mL, the range is adjusted to 1, 10, 20, 50 and 100 μg/mL. Additionally, when it is 5 μg/mL, the quantification range is set at 5, 10, 20, 50, and 100 μg/mL.
